# Draft Genome Sequence of Strain X47-2AL, a Feline *Helicobacter pylori* Isolate

**DOI:** 10.1128/genomeA.01095-13

**Published:** 2013-12-19

**Authors:** Frédéric J. Veyrier, Chantal Ecobichon, Ivo G. Boneca

**Affiliations:** Institut Pasteur, Biology and Genetics of the Bacterial Cell Wall Unit, Paris, Francea; Institut National de la Sante et de la recherche Medicale, Group Avenir, Paris, Franceb; Institut Pasteur, Invasive Bacterial Infections Unit, Paris, Francec

## Abstract

*Helicobacter pylori* is a human-specific pathogen that exclusively inhabits the human gastric mucosa. However, occasionally, humans transmit *H. pylori* to susceptible animal hosts bred in colonies. Here, we report the genome sequence of strain X47-2AL, isolated from a domestic cat and used in anti-*H. pylori* immunization studies.

## GENOME ANNOUNCEMENT

*Helicobacter pylori* strain X47-2AL was first isolated in 1995 from a colony of domestic cats which presented clinical signs of gastritis (1). The natural isolation of *H. pylori* strains in domestic cats but not from wild cats indicated that these were acquired by transmission from humans to cats. This is reminiscent of the recent emergence of *Helicobacter acinonyx* in big wild cats, probably from an ancestral *Helicobacter pylori* human hpAfrica2 strain (2). The first vaccination trials against *H. pylori* using recombinant urease were challenged with strain X47-2AL (3, 4). In fact, the strain is able to colonize the mouse model with a clear tropism for the corpus, in striking contrast to the mouse-adapted strain SS1, which preferentially colonizes the antrum. This work showed experimentally for the first time that two distinct strains can co-colonize the same stomach by occupying distinct microniches (5). In addition, X47-2AL is highly transformable, with DNA fragments allowing for efficient mutagenesis, although this strain does not take plasmid DNA. The strain has a resident plasmid incompatible with the shuttle vectors derived from pHeL2 and pHeL3 (6, 7). Additionally, the strain is naturally resistant to metronidazole, precluding the use of the *rdxA* locus for complementation studies.

Here, we announce the draft genome of the *H. pylori* strain X47-2AL. Genomic DNA was extracted using a QIAamp DNA minikit (Qiagen) from an overnight culture grown on blood agar plates. Whole-genome sequencing was performed using an Illumina HiSeq 2000 sequencer, which generated 50-bp paired reads. Furthermore, a large insert library was also sequenced (3-kb mate pair). The sequencing was done by GATC Biotech using standard protocols per the manufacturer’s instructions, which were followed during the sequencing process. The sequences (two million reads) were *de novo* assembled using the CLC Bio genomic suite with 78 contigs with an average length of 20 kb. The resulting contigs were subsequently scaffolded using a 3-kb mate pair library, to result in 63 contigs with an *N*_50_ of 53 kb and an average length of 25 kb. Contigs were submitted to the NCBI WGS submission portal and sequences were annotated using the NCBI Prokaryotic Genomes Automatic Annotation Pipeline (PGAAP) (http://www.ncbi.nlm.nih.gov/genomes/static/Pipeline.html). The genome comprises 1,495 coding sequences as well as a complete set of 36 tRNA and 3 rRNA (5S, 16S and 23S) coding loci. The average GC content of all contigs was 38.9%, which is identical to that of strain 26695 and very similar to the J99 (39.2%) and N6 (38.7%) genomes.

### Nucleotide sequence accession numbers.

This whole-genome shotgun project has been deposited at DDBJ/EMBL/GenBank under the accession number AWNG00000000. The version described in this paper is version AWNG01000000.
